# IL-17A impairs host tolerance during airway chronic infection by *Pseudomonas aeruginosa*

**DOI:** 10.1038/srep25937

**Published:** 2016-05-18

**Authors:** Nicola Ivan Lorè, Cristina Cigana, Camilla Riva, Ida De Fino, Alessandro Nonis, Lorenza Spagnuolo, Barbara Sipione, Lisa Cariani, Daniela Girelli, Giacomo Rossi, Veronica Basso, Carla Colombo, Anna Mondino, Alessandra Bragonzi

**Affiliations:** 1 Infections and Cystic Fibrosis Unit, Division of Immunology, Transplantation and Infectious Diseases, IRCCS San Raffaele Scientific Institute, Milano, Italy; 2 University Center for Statistics in the Biomedical Sciences (CUSSB), Vita-Salute San Raffaele University, Milano, Italy; 3 Cystic Fibrosis Microbiology Laboratory, Fondazione IRCCS Ca’ Granda, Ospedale Maggiore Policlinico, Milano, Italy; 4 School of Biosciences and Veterinary Medicine, University of Camerino, Italy; 5 Cystic Fibrosis Center, Fondazione IRCCS Ca’ Granda, Ospedale Maggiore Policlinico, Milano, Italy; 6 Lymphocytes Activation Unit, Division of Immunology, Transplantation and Infectious Diseases, IRCCS San Raffaele Scientific Institute, Milano, Italy

## Abstract

Resistance and tolerance mechanisms participate to the interplay between host and pathogens. IL-17-mediated response has been shown to be crucial for host resistance to respiratory infections, whereas its role in host tolerance during chronic airway colonization is still unclear. Here, we investigated whether IL-17-mediated response modulates mechanisms of host tolerance during airways chronic infection by *P. aeruginosa*. First, we found that IL-17A levels were sustained in mice at both early and advanced stages of *P. aeruginosa* chronic infection and confirmed these observations in human respiratory samples from cystic fibrosis patients infected by *P. aeruginosa*. Using *IL-17a*^−/−^ or *IL-17ra*^−/−^ mice, we found that the deficiency of IL-17A/IL-17RA axis was associated with: i) increased incidence of chronic infection and bacterial burden, indicating its role in the host resistance to *P. aeruginosa*; ii) reduced cytokine levels (KC), tissue innate immune cells and markers of tissue damage (pro-MMP-9, elastin degradation, TGF-β_1_), proving alteration of host tolerance. Blockade of IL-17A activity by a monoclonal antibody, started when chronic infection is established, did not alter host resistance but increased tolerance. In conclusion, this study identifies IL-17-mediated response as a negative regulator of host tolerance during *P. aeruginosa* chronic airway infection.

Two evolutionarily conserved host defense strategies to narrow disease severity have been described in host-pathogens interplay: resistance aims to contrast and eventually eradicate pathogenic bacteria, whereas tolerance limits the consequences of productive infections^1^,^2^,^3^. Altered mechanisms of resistance and tolerance can contribute to the aberrant inflammatory response during chronic airways diseases. In this context, persistent infections by *Pseudomonas aeruginosa* together with chronic inflammatory responses and progressive tissue damage are all hallmarks of chronic respiratory diseases, such as cystic fibrosis (CF) and advanced chronic obstructive pulmonary disease (COPD)^4^,^5^,^6^. The pathophysiological mechanisms that control host resistance and/or tolerance in chronic airways diseases remain to be deciphered.

Interleukin 17A (IL-17A) and IL-17 cytokines family have been suggested to participate to the pathogenesis of several respiratory diseases^7^,^8^,^9^,^10^,^11^,^12^. The IL-17-induced host response contributes to resistance mechanisms by playing a protective role at the mucosal barriers against pathogens such as *Staphylococcus aureus*, *Citrobacter rodentium* or *Klebsiella pneumonia*^13^. Recent data suggest that IL-17 pathway may play a key role in resistance and modulation of the inflammatory response during *P. aeruginosa* acute infection^14^,^15^,^16^,^17^. In addition, the IL-17-mediated host response has been shown to increase the secretion of matrix metalloproteinases (MMP)^18^, involved in tissue remodeling. All together these evidences suggest that type 17 immunity may be involved in the pathogenesis of chronic respiratory diseases, modulating both host resistance and tolerance.

Clinical proofs support the idea of a role for IL-17 in the pathogenesis of chronic respiratory diseases^12^. Indeed, IL-17 levels are elevated in several inflammatory lung diseases, such as CF and COPD^19^,^20^. In particular, in CF, IL-17 levels have been found to negatively correlate with Forced expiratory volume in 1 second (FEV1), suggesting its role in the decline of the lung function^21^. Among the potential cellular sources, IL-17 producing CD4^+^ T cells have been increasingly described in CF^22^. Thus, these data prompt the hypothesis that CF could be a IL-17-mediated disease^11^,^22^,^23^.

To date, the relative contribution of IL-17 to mechanisms of host resistance and tolerance during advanced chronic airways infections remains to be clarified^12^. Here, we addressed these issues in mice chronically infected for long term and in CF patients infected by *P. aeruginosa*. In the experimental murine model, we found that IL-17A levels were sustained over the course of *P. aeruginosa* chronic infection. In respiratory samples from CF patients, we confirmed that increased IL-17A levels were associated to both early and advanced stages of *P. aeruginosa* infection, strengthening the importance of the IL-17A-mediated response in the overall progression of chronic airways disease. Mechanistically, using *IL-17a*^−/−^ and *IL-17ra*^−/−^ mice, we demonstrated that the IL-17A/IL-17RA axis plays a dual role during chronic infection by *P. aeruginosa*: while it contributes to the host resistance, it weakens host tolerance, promoting immunopathology, during chronic airways infection. Moreover, targeting IL-17A when chronic infection is already established limits immunopathology, without compromising resistance to *P. aeruginosa* respiratory infection. Thus, our results indicate the role of IL-17A in modulating host tolerance during *P. aeruginosa* persistent infections and propose it as a potential target to modulate immunopathology in the context of chronic airways diseases.

## Results

### Host responses to *P. aeruginosa* during early and advanced chronic infection

**Figure 1:**
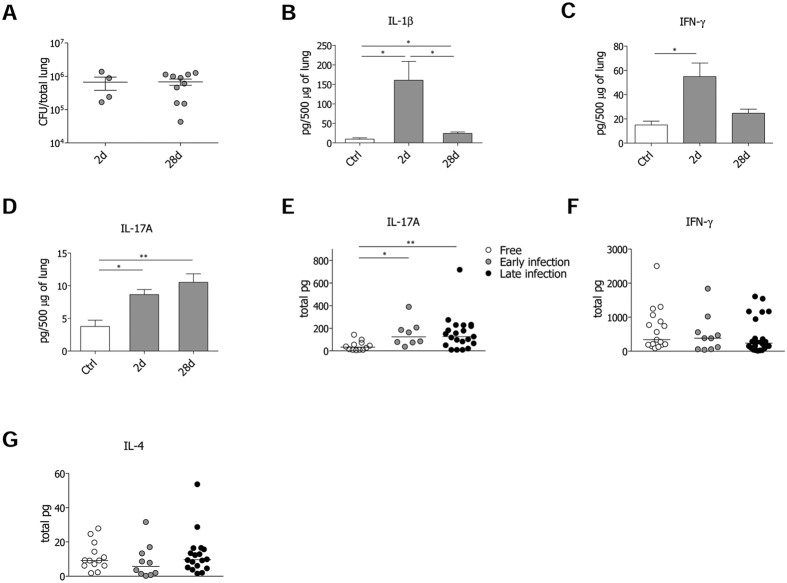
*P. aeruginosa* infection and markers of immune response in the murine model of chronic airways infection and CF patients. Two groups of minimum five C57Bl/6 mice were infected with 1 to 2 × 10^6^ CFU/lung of the *P. aeruginosa* strain AA43 embedded in agar beads and analyzed after 2 and 28 days of infection. Control (Ctrl) mice were treated with sterile agar-beads. (**A**) CFU were evaluated in total lung. Dots represent CFU in individual mice, horizontal lines represent mean values and the error bars represent the standard error of the mean (SEM). The data are pooled from at least two independent experiments (n = 4–12). Cytokines and chemokines, including IL-1β (**B**), IFN-γ (**C**), and IL-17A (**D**), were measured by Bioplex in murine lung homogenates. The data are pooled from at least two independent experiments (n = 3–9). Values represent the mean ± SEM. Levels of IL-17A (**E**), IFN-γ (**F**) and IL-4 (**G**) in sputa from CF patients never colonized by *P. aeruginosa* (free), with intermittent or chronic colonization up to two years (early) and with chronic colonization for at least four years (late) are compared. Dots represent values in individual patients and horizontal lines represent median values. Statistical significance is indicated: ^∗^p < 0.05, ^∗∗^p < 0.01.

While previous mechanistic studies in murine models attributed a role to IL-17 and IL-17 producing cells during acute early phases of *P. aeruginosa* infection^14^,^15^,^16^, here we focused on advanced *P. aeruginosa* chronic infection (28 days) in comparison with early acute phase (2 days). We adopted the *P. aeruginosa* AA43 isolate, which can establish chronic infection in C57Bl/6 mice with an incidence of colonization around 30–40%^24^,^25^. Cytokines typical of the immune response (IL-1β, IL-2, IFN-γ, IL-4, IL-17A) were analyzed at 2 and 28 days after infection. Although the bacterial burdens did not change among the early and advanced phases of infection (Fig. 1A), IL-1β levels significantly differed. IL-1β was acutely induced at day 2. By day 28, IL-1β levels had decreased, despite remaining significantly higher than those found in uninfected mice (Fig. 1B). While IL-4, indicative of type 2 immunity, was almost not detectable, IFN-γ, linked to type 1 immunity, was induced at the early stage and returned at basal levels at day 28 (Fig. 1C). Differently, IL-17A levels increased by day 2 and remained high over the course of *P. aeruginosa* infection (Fig. 1D).

Next, we analysed IL-17A, IFN-γ and IL-4 levels in sputa from patients with CF. The samples were collected from a cohort of 55 clinically stable CF patients recruited during routine care plans (Table S1), regardless of *P. aeruginosa* presence in the microbiological cultures. To evaluate whether IL-17A levels change according to the stage of *P. aeruginosa* infection, we distinguished CF patients into three categories: i) free, ii) “early” (intermittent *P. aeruginosa* infection and chronic colonization for up to two years) iii) “late” (chronic colonization for more than four years). IL-17A levels were significantly increased in both “early” and “late” patients in comparison to free patients (Fig. 1E), showing a similar trend to that observed in mice during *P. aeruginosa* chronic infection. As to IFN-γ and IL-4, their levels did not differ (Fig. 1F,G). Moreover the sera of CF patients colonized by *P. aeruginosa* revealed low or undetectable levels of IL-17A (Table S2), suggesting IL-17A release to be restricted to the lung compartment rather than systemic. Thus, while type 1 and 2 immunities do not seem to participate to the advanced stages of *P. aeruginosa* infection in the lung compartment, IL-17A-mediate response might play a critical role during the advanced stage of chronic infection in both humans and mice.

### Type 17 immunity is sustained during *P. aeruginosa* chronic infection

**Figure 2:**
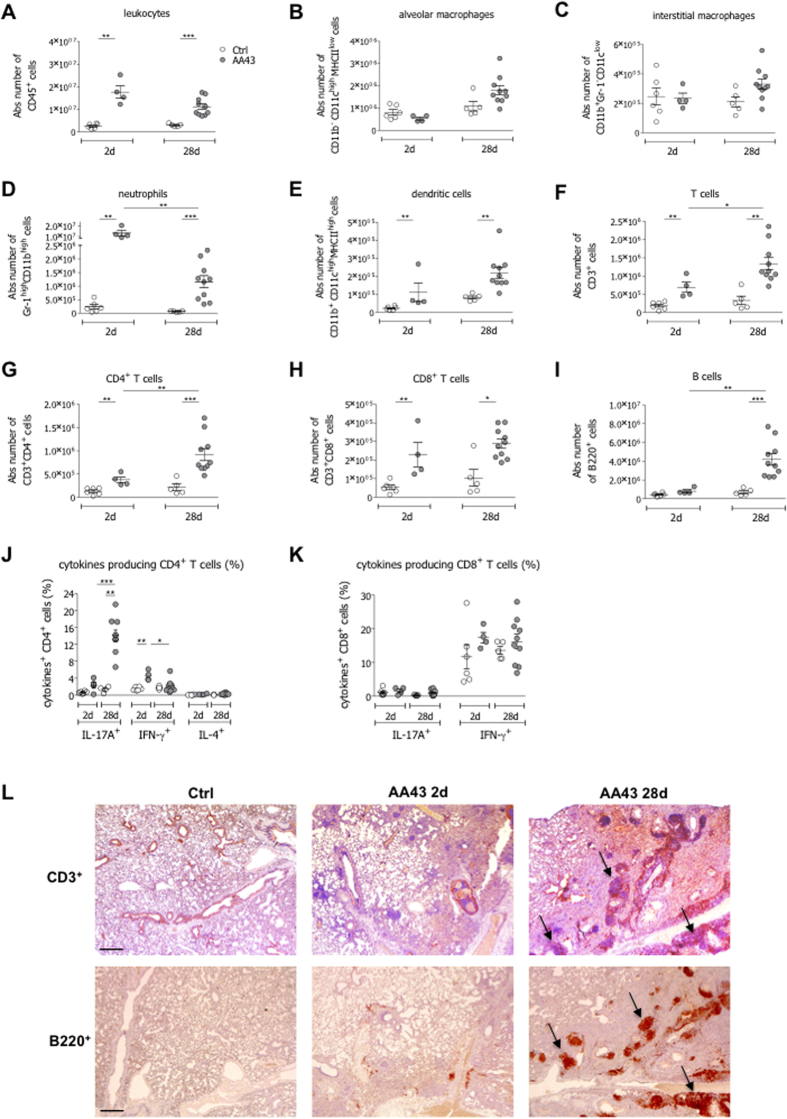
Immune response in the murine model of chronic airways infection with *P. aeruginosa*. C57Bl/6 mice were infected with 1 to 2 × 10^6^ CFU/lung of the *P. aeruginosa* strain AA43 embedded in agar beads and analyzed after 2 and 28 days of infection. Ctrl mice were treated with sterile agar-beads. The absolute numbers of leukocytes (**A**), alveolar macrophages (**B**), interstitial macrophages (**C**), neutrophils (**D**), dendritic cells (**E**), T cells (**F**), CD4^+^ T cells (**G**), CD8^+^ T cells (**H**) and B cells (**I**) were measured by flow cytometric analysis in cell suspensions of murine lungs. The frequency of IL-17A-, IFN-γ- and IL-4-producing CD4^+^ T (**J**) and CD8^+^ T (**K**) cells were measured in lung cell suspensions after PMA/ionomycin stimulation, by flow cytometric analysis. The data are pooled from at least two independent experiments (n = 4–12). Dots represent cells in individual mice, horizontal lines represent mean values and the error bars represent the SEM. Statistical significance is indicated: *p < 0.05, **p < 0.01, ***p < 0.001. (**L**) Lung immunohistochemistry was performed on challenged mice by staining with anti-CD3 and anti-B220 antibodies. Scale bars: 400 μm. Some BALT-like structures are indicated by arrows.

Lung infiltrating cells were further dissected by Fluorescence-activated cell sorting (FACS) analysis and immunohistochemistry in murine models. The number of infiltrating leukocytes was significantly higher in samples recovered from infected mice at both day 2 and 28 post-infection when compared to controls (Fig. 2A). While alveolar and interstitial macrophages were not enriched for, both neutrophils and dendritic cells (DC) were accumulated in infected lungs. However, neutrophils counts decreased by day 28, whereas DC numbers remained high at the advanced phase of *P. aeruginosa* chronic infection (Fig. 2B–E). Also T and B cell representations were significantly different in control and infected mice. While T cells, comprising both CD4^+^ and CD8^+^ subsets, were significantly enriched for both at day 2 and day 28 and CD4^+^ subsets further increased at day 28 (Fig. 2F–H), B cell numbers were significantly higher only at the advanced stage of chronic infection (Fig. 2I). This was in agreement with a selective enrichment of CD4^+^ IL-17A^+^ T cell, but not of CD4^+^ IL-4^+^ and CD4^+^ IFN-γ^+^ T cells (Fig. 2J). Differently, CD8^+^ T cells capable of IFN-γ or IL-17A did not change over the course of chronic *P. aeruginosa* infection (Fig. 2K) and those secreting IL-4 were not detectable. By immunohistochemistry we found that T and B cells co-localized in bronchus-associated lymphoid tissue (BALT)-like structures at day 28 (Fig. 2L), likely indicating an active adaptive immune response in the airways. The distribution of cytokine-secreting CD4^+^ T cells did not differ in the spleen of infected and not infected mice (Table S3), supporting the existence of tissue-restricted events in *P. aeruginosa*-infected mice. Together these data highlight the potential impact of type 17 immunity in the airways disease progression during *P. aeruginosa* chronic infection.

### Depletion of the IL-17A/IL-17RA axis reduces host resistance while favoring tolerance to *P. aeruginosa* infection

**Figure 3:**
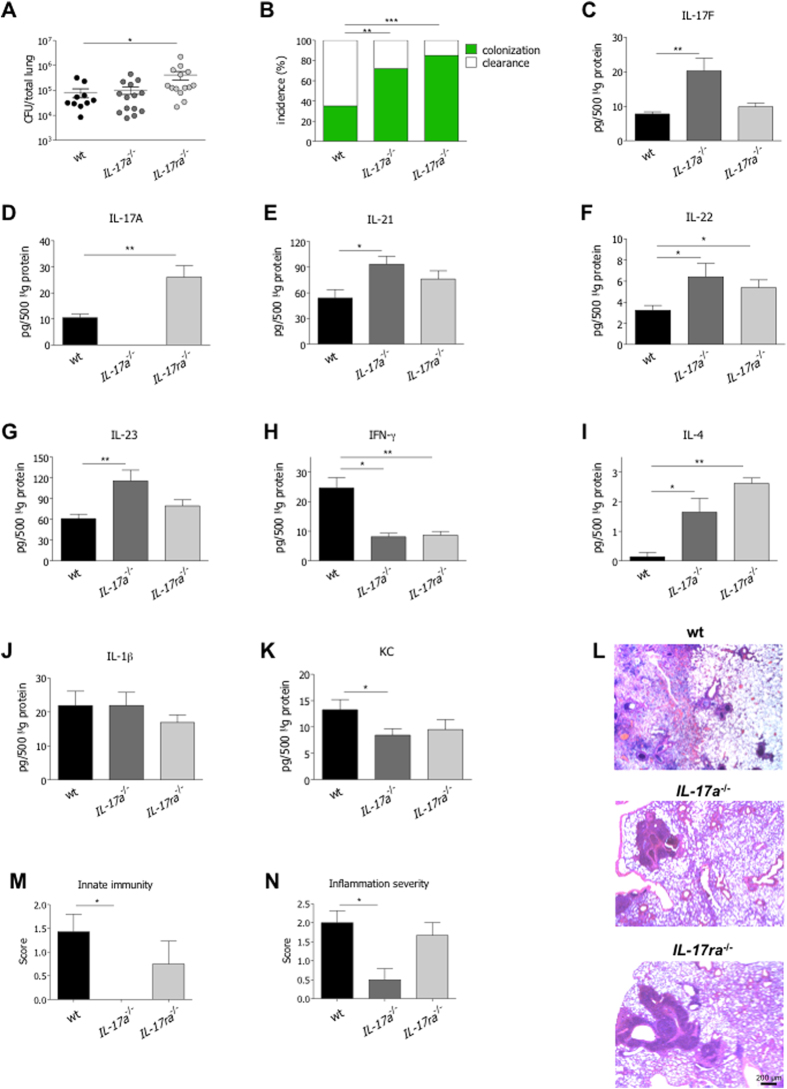
Virulence of *P. aeruginosa* and host immune response to chronic airways infection in *IL-17a*^*−/−*^, *IL-17ra*^*−/−*^ and congenic wt mice. Two groups of minimum five *IL-17a*^−/−^, *IL-17ra*^−/−^ and congenic wt C57Bl/6 mice were infected with 1 to 2 × 10^6^ CFU/lung of the *P. aeruginosa* strain AA43 embedded in agar beads and analyzed 28 days post-infection. Ctrl mice were treated with sterile agar-beads. (**A**) CFU were evaluated in total lung. Dots represent CFU in individual mice, horizontal lines represent mean values and the error bars represent the SEM. The data are pooled from at least two independent experiments (n = 10–14). (**B**) Bacterial clearance (white) and incidence of colonization (green) were determined. The data are pooled from at least two independent experiments (n = 17–29). Cytokines and chemokines, including IL-17F (**C**), IL-17A (**D**), IL-21 (**E**), IL-22 (**F**), IL-23 (**G**), IFN-γ (**H**), IL-4 (**I**), IL-1β (**J**) and KC (**K**), were measured by Bioplex in lung homogenates of mice after 28 days of chronic lung infection with *P. aeruginosa*. The data are pooled from at least two independent experiments (n = 3–14). Values represent the mean ± SEM. Sections of airways from mice infected with AA43 for 28 days were stained with H&E, according to the standard procedure (**L**). Scale bars: 200 μm. Innate immune cells infiltration (**M**) and inflammation severity (**N**) were scored in tissue sections. Values represent the mean ± SEM. The data are pooled from two independent experiments (n = 3–5). Statistical significance is indicated: *p < 0.05, **p < 0.01, ***p < 0.001.

To directly establish the role of the IL-17A/IL17-RA axis in host resistance and/or tolerance to *P. aeruginosa* colonization, we infected *IL-17a*^−/−^ and *IL-17ra*^−/−^ in comparison with wt congenic mice. Bacterial burden, inflammation and tissue damage were evaluated at day 28 after infection. *P. aeruginosa* lung infection did not lead to mortality in *IL-17a*^−/−^ and *IL-17ra*^−/−^ mice, similarly to wt mice (data not shown). Higher bacterial load was observed in *IL-17ra*^−/−^ but not in *IL-17a*^−/−^ mice (Fig. 3A). However, the incidence of colonization was increased in both *IL-17a*^−/−^ and *IL-17ra*^−/−^ mice (Fig. 3B). Overall, these results suggest that host resistance to *P. aeruginosa* infection can be principally ascribed to the IL-17RA downstream signaling, rather than to IL-17A alone.

By studying cytokines/chemokines profile in lung homogenates, we found higher levels of IL-17F in *IL-17a*^−/−^ mice, and of IL-17A in *IL-17ra*^−/−^ mice, likely due to compensatory mechanisms (Fig. 3C,D). In addition, we also found higher levels of type 17 cytokines, such as IL-21, IL-22 and IL-23 in *IL-17a*^−/−^ mice, and comparable trends in *IL-17ra*^−/−^ mice (Fig. 3E–G). Of note, while IFN-γ levels decreased in deficient mice, IL-4 levels were significantly increased (Fig. 3H,I). IL-1β levels were comparable between wt and deficient mice (Fig. 3M). Of note, KC levels were significantly reduced in *IL-17a*^−/−^ mice, with a similar trend in *IL-17ra*^−/−^ mice (Fig. 3K) despite a higher bacterial load. Accordingly, the number of lung infiltrating neutrophils and macrophages as representative of the innate immune response, and the inflammation severity, in terms of infiltrating inflammatory cells combined to alveolar damage, were significantly reduced in *IL-17a*^−/−^ mice, with a similar trend in *IL-17ra*^−/−^ mice (Fig. 3L,M). Thus, deficiency of the IL-17A/IL-17RA axis modulates the immune response, regardless of the bacterial burden, suggesting a potential role of the axis in mediating tolerance mechanisms.

**Figure 4:**
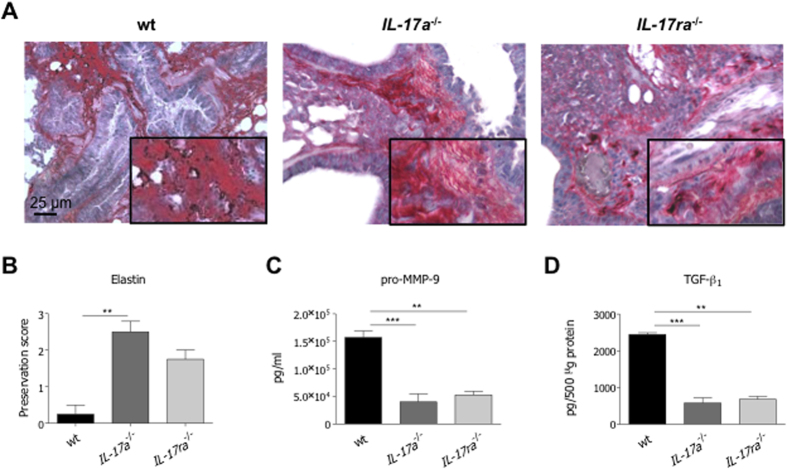
Tissue damage after *P. aeruginosa* chronic airways infection in *IL-17a*^−/−^, *IL-17ra*^−/−^ and congenic wt mice. Two groups of minimum five *IL-17a*^−/−^, *IL-17ra*^−/−^ and congenic wt C57Bl/6 mice were infected with 1 to 2 × 10^6^ CFU/lung of the *P. aeruginosa* strain AA43 embedded in agar beads and analyzed 28 days post-infection. Ctrl mice were treated with sterile agar-beads. (**A**) Sections of airway from mice infected with AA43 were stained with VEG for elastic fibers, according to the standard procedure. Scale bars: 25 μm. Scoring of elastin preservation was performed on slices stained with VEG (**B**). Levels of pro-MMP-9 (**C**) and TGF-β_1_ (**D**) were measured by ELISA and Bioplex respectively in lung homogenates after 28 days of chronic lung infection. Values represent the mean ± SEM. The data are pooled from at least two independent experiments (n = 3–10). Statistical significance is indicated: *p < 0.05, **p < 0.01, ***p < 0.001.

Given observed differences, we quantified markers associated to tissue damage trying to prove the role of IL-17-mediated response in the modulation of host tolerance mechanisms. Elastin degradation, levels of pro-MMP-9 and TGF-β_1_ were significantly reduced in *IL-17a*^−/−^ mice (Fig. 4A–D). Similar trends were also observed in *IL-17ra*^−/−^ mice, confirming that deficiency of the IL-17A/IL-17RA axis is associated to tissue preservation. Thus, together the data indicate that chronic IL-17-induced responses propagate local immune manifestations and induce tissue damage in response to persistent *P. aeruginosa* infection by hindering tolerance mechanisms.

### IL-17A targeting improves tolerance during *P. aeruginosa* chronic airways infection

**Figure 5:**
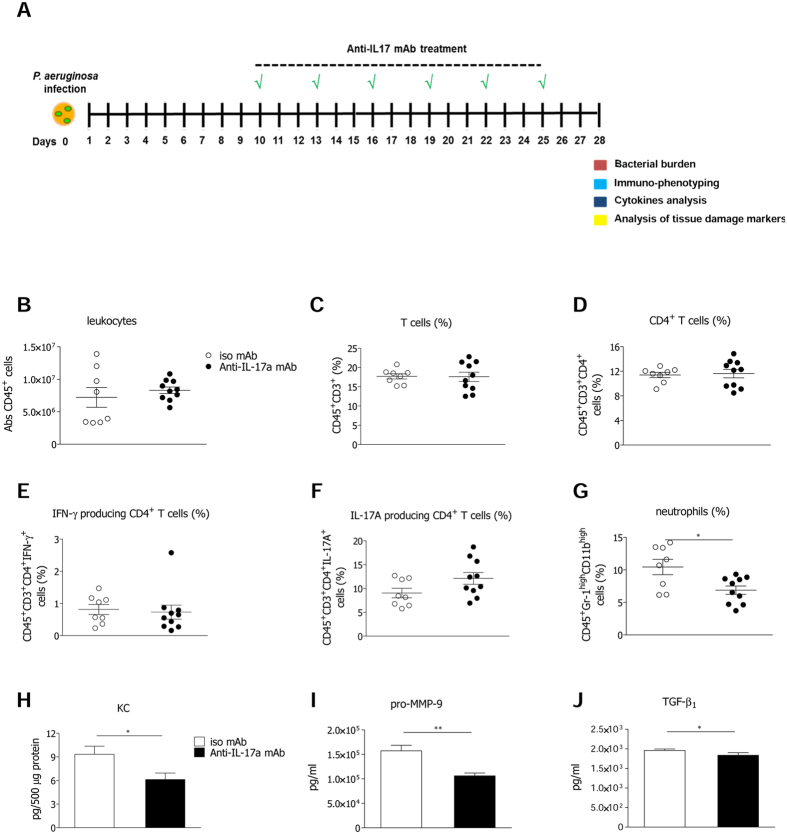
Anti-IL-17A mAb treatment in the murine model of chronic airways infection by *P. aeruginosa*. C57Bl/6 mice were infected with 1 to 2 × 10^6^ CFU/lung of the *P. aeruginosa* strain AA43 embedded in agar beads. Every three days starting from the tenth day from infection, a group of mice was treated by intraperitoneal injection (i.p.) with 100 μg/mouse of anti-IL-17A mAb, while the other group was treated with 100 μg/mouse of control isotypic IgG and analyzed after 28 days of infection. Schematic view of the treatment schedule is shown (**A**). Leukocytes (**B**), T cells (**C**), CD4^+^ T cells (**D**), the frequency of IFN-γ- (**E**) and IL-17A-producing CD4^+^ T cells (**F**) after PMA/ionomycin stimulation, and neutrophils (**G**) were measured by flow cytometric analysis in cell suspensions of murine lungs. Dots represent cells in individual mice, horizontal lines represent mean values and the error bars represent the SEM. KC levels (**H**) were measured by Bioplex in the supernatants of murine lung cellular suspensions. Levels of pro-MMP-9 (**I**) by ELISA and TGF-β_1_ (**J**) by Bioplex were measured in the supernatants of murine lung cellular suspensions. Values represent the mean ± SEM. The data are pooled from at least two independent experiments (n = 8–10). Statistical significance is indicated: ^*^p < 0.05, ^**^p < 0.01.

Next, we investigated whether targeting the IL-17A/IL-17RA axis rescues tolerance mechanisms during *P. aeruginosa* chronic infection. Taking into consideration the noxious effect of *IL-17ra* deficiency on host resistance, in term of bacterial burden, we adopted blocking anti-IL-17A mAb. Schedule of treatment with anti-IL-17A mAb was set-up from 10 days post infection to distinguish IL-17A contributions to the lung pathology during advanced *P. aeruginosa* chronic infection (Fig. 5A) from those determined at the early/acute phase of infection in *IL-17a*^−/−^ mice. At day 28, mice treated with isotype IgG control and anti-IL-17A mAb showed comparable bacterial load and colonization incidence (Fig. S1A). Also leukocyte (interstitial macrophages, alveolar macrophages, B cells and cytotoxic T cells) (Fig. S1B–E) and T cell numbers, along with CD4^+^ T cells, IL-17A and IFN-γ producing CD4^+^ T cells were comparable in isotype IgG and anti-IL-17A-treated mice (Fig. 5B–F), while IL-4 producing CD4^+^ T cells were undetectable. Moreover, IL-17A and IFN-γ levels were comparable in mice treated with isotype IgG control and anti-IL-17A mAb (data not shown). Nevertheless, we found that IL-17A blockade decreased neutrophils infiltration (Fig. 5G), likely due to reduced KC levels (Fig. 5H). Remarkably, mice treated with anti-IL-17A mAb displayed also lower levels of pro-MMP-9 (Fig. 5I) and TGF-β_1_ (Fig. 5J), indicating lower production of markers promoting tissue remodeling and damage. Results suggest that IL-17A neutralization does not provoke bacterial spreading, and instead promotes host tolerance mechanisms, narrowing exacerbated pulmonary neutrophilia and tissue damage associated with chronic *P. aeruginosa* airways infection.

## Discussion

The mechanisms underlying the exaggerated inflammation and tissue damage, associated to un-resolved airways infections, in CF^4^ and others chronic lung diseases, such as COPD^26^,^27^, are thought to be associated with an inappropriate host response. Here we demonstrate that type 17 immunity is central to the exaggerated and detrimental host response to *P. aeruginosa* chronic infection. Indeed, while previous data indicated a role for IL-17A in resistance to microbial insults in the acute/early phases of infection^12^, our results underline its involvement in impairing host tolerance during advanced chronic *P. aeruginosa* airways infection.

Using mouse models of short term infection by *P. aeruginosa*, previous studies demonstrated that IL-17 and IL-17^+^ cells contribute to the host response in the acute phase, mediating neutrophils and T cells recruitment^10^. During the early/acute phases of *P. aeruginosa* infection, γδ T or Type 3 pulmonary Innate Lymphoid Cells (pILC3) cells were found to be the main cellular sources of IL-17 cytokines^12^,^16^,^28^. To investigate the previously unexplored role for IL-17A during advanced chronic *P. aeruginosa* airways infection we took advantage of the agar-beads mouse model^25^,^29^, and characterized innate and adaptive immune responses at the early and advanced phases of infection. We found that type 1 and type 17 cytokine responses could all be detected at early phases of infection. However, while IFN-γ cytokines returned to background levels in chronically infected mice, IL-17A levels remained high. In support of our experimental evidences, we found elevated IL-17A levels in CF human respiratory samples at both early and late phases of *P. aeruginosa* infection, fostering the concept that the type 17 immunity is involved during the overall course of chronic infection by *P. aeruginosa.*

Next, we dissected the inflammatory response by FACS analysis. We proved that CD4^+^ IL-17A^+^ cells were greatly increased, suggesting their involvement in chronic inflammation, while we did not find neutrophils secreting IL-17A (data not shown) at the advanced stage of infection. However, we cannot exclude that other cellular sources (ILCs or γδ T cells) may be involved during *P. aeruginosa* long-term chronic infection in our mouse model. Further studies are needed to explore this possibility. In addition to T cells, B cells and DC were found in association to the presence of BALT like-structure, likely indicative of active local responses. Interestingly, the absence of IL-17A^+^ T cells in murine spleens indicated that T cell differentiation in response to pathogenic insults is tissue specific rather than systemically activated, supporting other studies on chronic lung diseases (e.g. severe COPD)^30^.

The role of IL-17 in protective versus detrimental immunity has been debated^8^,^31^. Recent evidences indicate that the IL-17 pathway exerts a protective effect in the airways by favoring the clearance of several pathogens^8^,^32^. IL-17A and IL-17F cytokines may have a redundant function in host defense through the heterodimeric receptor complex formed by IL-17RA and IL-17RC^33^. To validate the hypothesis that IL-17A-mediated response may have a role in modulating host resistance during chronic *P. aeruginosa* infection, *IL-17a* and *IL-17ra* deficient mice were explored. Moreover, we took advantage of a *P. aeruginosa* isolate, namely AA43, known to establish stable chronic infection in the 30–40% of challenged mice^24^,^25^. This choice was fundamental to uncover that the incidence of chronic colonization was increased in both *IL-17a* and *IL-17ra* deficient mice when compared to wt mice. Differently, bacterial burdens were altered only by *IL-17ra* deficiency. This suggests that host resistance to *P. aeruginosa* is mediated by IL-17A and IL-17F, through IL17RA, rather than only by IL-17A.

In addition to resistance, several evidences suggested the IL-17 pathway as detrimental to chronic inflammatory airways diseases^34^,^35^. Indeed, blockade of IL-17A in bleomycin-induced acute inflammation promoted the resolution of inflammation^36^. IL-17 was associated with airways hyperresponsiveness and to increased mucus production in murine model of respiratory syncytial virus disease or allergic disease^31^,^37^. Other studies demonstrated the contribution of IL-17A in remodeling processes, such as the development of chronic fibroproliferative disease^38^. Expanding this notion in the context of chronic inflammatory processes mediated by persistent *P. aeruginosa* infection, our findings showed that IL-17A pathway affects the host tolerance, increasing leukocytes recruitment and causing tissue damage. Although the bacterial burden did not change in *IL-17a*, but even increased in the *IL-17ra* deficient mice, markers of tissue remodeling and damage, such as elastin degradation, and levels of pro-MMP-9 and TGF-β_1_ were decreased. Thus, our findings demonstrated that prolonged local release of IL-17A affects host tolerance favoring the development of excessive immunopathology during chronic *P. aeruginosa* infection.

Experimental evidences of a detrimental role of IL-17A in chronic *P. aeruginosa* infection prompted us to evaluate whether neutralizing IL-17A might provide a valuable strategy in the effort of limiting pathogen-associated tissue damage, without altering host resistance. To avoid an exacerbation of bacterial lung burden in the early phase of infection^39^,^40^, we decided to interfere with IL-17A, leaving the IL-17F and IL-17RA able to operate during the advanced stage of chronic infection. We found that anti-IL-17A mAb treatment did not increase the bacterial burden, while it significantly reduced local inflammatory response and tissue remodeling and damage. Interestingly, the finding that IL-17A targeting partially reduced neutrophils infiltration and did not affect other immune cells suggests that IL-17F may have a complementary function in modulating immune response, as previously described for other pathogens^8^,^13^. Given the notion that IL-17 contributes to the pathogenesis of several chronic diseases and inhibitors have been identified^12^,^34^, it is tempting to speculate that IL-17A might be a critical target in *P. aeruginosa* infected patients, in particular those chronically infected, to ameliorate their lung function.

In conclusion, our work has elucidated the role of IL-17A during chronic airways infection by *P. aeruginosa* and may provide the ground for designing novel immunotherapy strategies, modulating mechanisms of resistance and tolerance. We indeed proved that IL-17-mediated immunity plays a double-edged activity during chronic airways infection: on one side, it contributes to the control of *P. aeruginosa* burden, modulating host resistance, while, on the other, it alters host tolerance, propagating exacerbated pulmonary neutrophilia and tissue remodeling. Inhibition of the IL-17 pathway in a time-specific fashion represents a novel potential host-based intervention to ameliorate lung physiology and function without compromising host resistance against pathogens during chronic airways infection.

## Methods

### Ethics statement

Animal studies were conducted according to protocols approved by the San Raffaele Scientific Institute (Milan, Italy) Institutional Animal Care and Use Committee (IACUC) and adhered strictly to the Italian Ministry of Health guidelines for the use and care of experimental animals (IACUC protocol #505), following the guidelines included in the D. Lgs. 04-03-2014 n. 26. Research with AA43 bacterial strain, used for animal experiments, has been approved by the Ethics Commission of Hannover Medical School, Germany. The patient and parents gave informed consent before the sample collection. Approval for storing of biological materials was obtained by the Ethics Commission of Hannover Medical School, Germany. The study on human samples from the Regional CF Center of Lombardia was approved by the Ethical Committees of San Raffaele Scientific Institute and Fondazione IRCCS Ca’ Granda, Ospedale Maggiore Policlinico, Milan, Italy, and written informed consent was obtained from patients enrolled or their parents according to the Ethical Committee rules, in accordance with the laws of the Italian Ministero della Salute (approval #1874).

### Bacterial strains

The *P. aeruginosa* isolate, AA43, from a CF patient, was chosen from a strain collection, previously characterized for genotypic and phenotypic traits and virulence^24^,^25^,^41^.

### Mouse strains and model of chronic *P. aeruginosa* infection

C57Bl/6 (Charles River), *IL-17a*^−/−^ and *IL-17ra*^−/−^ mice (background C57Bl/6), male, 8–10 weeks old, were maintained in specific pathogen-free conditions. Mice were injected intratracheally with 1–2 × 10^6^ CFU of *P. aeruginosa*, embedded in agar beads, following established procedures^25^,^29^.

### Lung dissociation and processing for analysis of cytokines and markers of tissue damage

Mice were sacrificed by CO_2_ administration. Lungs were removed and homogenized^29^. Samples were plated for CFU count. Recovery of >1000 CFU from lung cultures was indicative of chronic infection. Lung homogenates were then centrifuged at 14000 rpm for 30 min at 4 °C and the supernatants (SN) were stored at −80 °C for quantification of total protein content with Bradford’s assay (Bio-RAD) and analysis of inflammatory and tissue damage markers.

### Flow cytometry and intracellular cytokines staining

Mechanical dissociation was used to prepare single cells suspensions from lung and spleen. Antibody staining was performed as previously described^42^,^43^. Briefly, after Fc blocking treatment, cells harvested from lungs and spleen were washed and stained for 20 minutes with appropriate dilutions of antibodies from the antigen-presenting cell (APC) panel and lymphoid cells (T cell) panel (Table 1) in FACS staining buffer (PBS, 2% FBS, 0, 2% NaN_3_). Then cells were washed twice, fixed (PBS 4% formaldehyde) and resuspended in FACS staining buffer. For intracellular staining, cells harvested from lungs and spleens were washed, incubated 2 hours with phorbol 12-myristate 13-acetate (PMA, 10 ng/ml, Sigma) and Ionomycin (1 μg/ml, Sigma), then brefeldin A (BFA) was added (5 μg/ml, Sigma). After 2 hours, samples were washed with FACS staining buffer, and, after Fc blocking treatment, cells were incubated with appropriate dilutions of antibodies from intracellular lymphoid cells panel (Table 1). Cells were washed twice. Fixation and permeabilization for cytokine intracellular staining were performed according to the manufacturer’s instruction. Anti-IL-17A, anti-IL-4 and anti-IFN-γ antibodies were used. For each antibody, a control isotype was used for compensation. Doublets were removed by gating on a plot of forward-scatter area versus forward-scatter height. Acquisition and analyses were performed using FACSCanto cytometer (BD Biosciences) and FlowJo Software (Tree Star).

### Inhibitory treatment in mice

Rat anti–IL-17A monoclonal antibody (Cat.# 506923) and isotype control were purchased from Biolegend. Animals were treated every 3 days with intraperitoneal (i.p.) injection of 100 μg/ mouse of antibodies starting 10 days post infection, when chronic airways infection was stably settled^44^.

### Histological examination

Murine lungs were removed, fixed in formalin, and embedded in paraffin. Consecutive sections from the middle of the five lung lobes were used for histological and immunohistochemical examination. Sections were collected and stained by anti-CD3 mAb or anti-B220 mAb, Haematoxylin and Eosin (H&E) and Verhoeff’s elastic (VEG) staining and were examined blindly and scored by a pathologist. Histological score analysis of murine lungs was performed to grade the amount of innate immune cells infiltration (macrophages and neutrophils) and inflammation severity (evaluating both innate and adaptive immune cells recruitment, and the extent of alveolar damage). Histological examination primarily scored the number of immune cells (mononuclear cells, such as macrophages, lymphocytes, plasma cells, and neutrophils) at a magnification of ×400. The results are reported as the mean for the entire specimen^45^. Immune cells were classified as absent (score of 0) when there were no or fewer than 19 cells per high-power field (HPF) (at a magnification of ×400), mild (score of 1) for 20 to 49 cells per HPF, moderate (score of 2) for 50 to 99 cells per HPF, marked or severe (score of 3) for 100 to 200 cells or more per HPF. Histological criteria for normal pulmonary characteristics included detection of no or only a few mononuclear cells per HPF and no or only a few scattered neutrophils in bronchioli and alveoli without tissue changes (no interstitial thickening or aggregates of lymphocytic infiltrates, and airways free from exudate). The number of inflammatory cells, assessed at ×400 and ×100 magnification respectively, was scored and customized as described by Martino *et al.*^46^.

VEG was used for assessing and quantifying the elastin architecture in lung interstitial and peribrochial areas. The degree of fragmentation and the amount of the elastic fibers were examined by the pathologist, blinded to the treatment group, who scored each slide using an arbitrary combined scoring system that counted the number of “islands of damage” within a lung cross-section from each mouse. An island of damage was defined as an isolated area of lung’s interstitium or peribronchus, where two adjacent elastic fibers were fragmented with interposed excessive connective tissue matrix, evaluated randomly on the cross-section at 400x. Three evaluations/lung were performed. The method was extrapolated from McLoughlin *et al.*^47^.

### CF patients and study design

Sputum was recovered from 55 CF patients at the regional CF centre of Lombardia (Fondazione IRCCS Ca’ Granda – Ospedale Maggiore Policlinico, Milan, Italy).

Inclusion criteria were: a) diagnosis after neonatal screening or after symptoms onset and followed since birth in the centre; b) complete clinical history available in the computerized database; c) at least two visits yearly since diagnosis; d) availability of at least four yearly sputum cultures for microbiological ascertainment; e) at least one CT every year; f) at least one respiratory function test/year after the age of 5 years.

Exclusion criteria were i) positive culture for *Burkholderia cepacia complex*, methicillin-resistant *Staphylococcus aureus, Achromobacter xylosoxidans*, *Stenotrophomonas maltophilia*, *Scedosporium* spp, and ii) acute pulmonary exacerbation (APE). APE was determined by the presence of at least four of the following criteria: 10% or greater decrease in baseline FEV_1_, increased cough or sputum production, change in sputum character, dyspnea, tachypnea, fever, weight loss, 5% or greater decrease in O_2_ saturation, new or worsening crackles on lung auscultation, or findings on chest X-ray consistent with pneumonia. The clinical procedures were applied for usual care plans. Data on age, gender, *cftr* genotype (ΔF508 homozygous) *P. aeruginosa* status and years of colonization, and presence of pathogens other than *P. aeruginosa,* were collected. The characteristics of the CF patients are summarized in the Table S1.

### CF patients sputum processing

Samples used to evaluate immune markers diluted in PBS to a final volume of 5 ml and they were centrifuged at 350 g for 10 min at 4 °C and SN collected and stored at −80 °C.

### Human respiratory samples microbiology

Respiratory samples were plated on chocolate agar with bacitracine, Columbia CNA Agar with 5% Sheep Blood, Mannitol salt agar, MacConkey II Agar, Cepacia Medium Agar and Sabouraud Dexstrose Agar. Microbic species were identified with MicroScan WalkAway plus System.

### Evaluation of markers associated to tissue damage and inflammation

Multiplex immunoassays (Bio-Rad) based on Luminex technology were used for the quantification of cytokines, chemokines and growth factors in murine and human samples, according to the manufacturer’s instruction. A mouse Bio-Plex custom mix was used to analyze KC, IL-1β, IL-4, IL-17A, IL-17F, IL-21, IL-22, IL-23, and IFN-γ in murine lungs. A human Bio-Plex custom mix was used to analyze IL-4, IL-17A, IFN-γ. The three isoforms of TGF-β (TGF-β_1_, TGF-β_2_ and TGF-β_3_) were analyzed with Bio-Plex Pro^TM^ TGF-β 3-plex panel in murine lungs. Data were measured on Bio-Plex 200 System and calculated using Bio-Plex Manager 6.0 and 6.1 software. Murine pro-MMP-9 in the lungs was measured by ELISA (R&D DuoSet ELISA Development System), according to the manufacturer’s instructions.

### Statistics

Statistics were performed with GraphPad Prism and R environment for statistical computing. When comparing data at a specific time-point a nonparametric two-taled Mann-Whitney U test was performed. To compare data in different murine strains and human samples in comparing groups of patients at different stage of infection (“free”, “early” and “late”) a nonparametric Kruskal-Wallis test was used followed by post-hoc Dunn test to correct for multiple comparisons. Incidences of colonization were compared using Fisher exact test. Statistical analyses were considered significant at p < 0.05.

## Additional Information

**How to cite this article**: Lorè, N. I. *et al.* IL-17A impairs host tolerance during airway chronic infection by *Pseudomonas aeruginosa. Sci. Rep.* **6**, 25937; doi: 10.1038/srep25937 (2016).

## Supplementary Material

Supplementary Information

## Figures and Tables

**Table 1:** Antibodies used for FACS analysis and intracellular staining are indicated.

APC antibodies panel	T cells antibodies panel	Intracellular T cells panel
anti-CD45	anti-CD45	anti-CD3
anti-GR-1	anti-CD3	anti-CD4
anti-CD11b	anti-CD4	anti-CD8
anti-CD11c	anti-CD8	anti-IL-17A
anti-MHCII	anti-CD44	anti-IFN-γ
anti-B220		anti-IL-4
