# Supplementary material for: IL-17A impairs host tolerance during airway chronic infection by Pseudomonas aeruginosa
Source: Sci Rep. 2016 May 18;6:25937. doi: 10.1038/srep25937 (PMC4870500; doi:10.1038/srep25937)
Supplement: Supplementary Information [file srep25937-s1.pdf]

IL-17A impairs host tolerance during airway chronic infection by *Pseudomonas aeruginosa*

Nicola Ivan Lorè[1 ¥ *], Cristina Cigana[1 ¥], Camilla Riva[1], Ida De Fino[1], Alessandro Nonis[2], Lorenza Spagnuolo[1], Barbara Sipione[1], Lisa Cariani[3], Daniela Girelli[3], Giacomo Rossi[4], Veronica Basso[6], Carla Colombo[5], Anna Mondino[6], Alessandra Bragonzi[*]

[1] Infections and Cystic Fibrosis Unit, Division of Immunology, Transplantation and Infectious Diseases, IRCCS San Raffaele Scientific Institute, Milano, Italy

[2] University Center for Statistics in the Biomedical Sciences (CUSSB), Vita-Salute San Raffaele University, Milano, Italy

[3] Cystic Fibrosis Microbiology Laboratory, Fondazione IRCCS Ca' Granda, Ospedale Maggiore Policlinico, Milano, Italy

[4] School of Biosciences and Veterinary Medicine, University of Camerino, Italy

[5] Cystic Fibrosis Center, Fondazione IRCCS Ca' Granda, Ospedale Maggiore Policlinico, Milano, Italy

[6] Lymphocytes Activation Unit, Division of Immunology, Transplantation and Infectious Diseases, IRCCS San Raffaele Scientific Institute, Milano, Italy

SUPPLEMENTARY INFORMATION

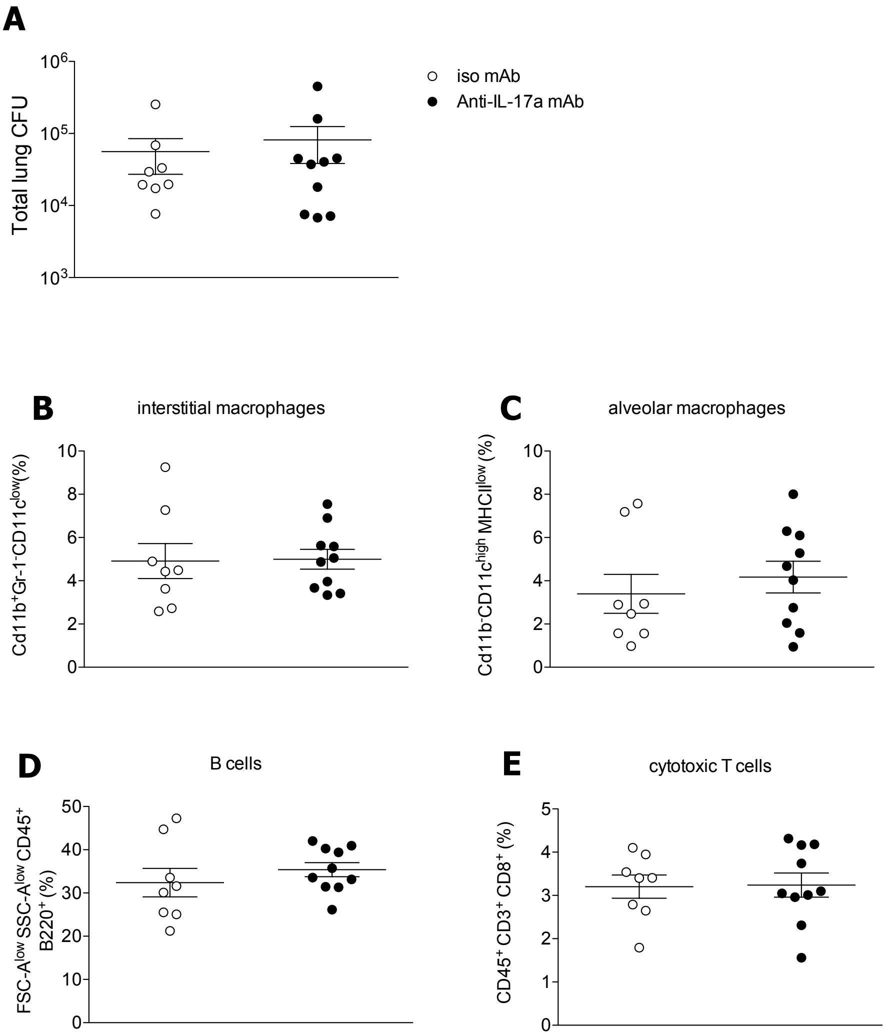

Fig. S1. *P. aeruginosa* bacterial load and host response in the lung after anti-IL-17A mAb treatment in the murine model of chronic airways infection. C57Bl/6NCrlBR mice were infected with 1 to 2x10^6 CFU/lung of the *P. aeruginosa* strain AA43 embedded in agar beads. Every three days starting from the tenth day from infection, a group of mice was treated i.p. with 100µg/mouse of anti-IL-17A mAb, while the other group was treated i.p. with 100µg/mouse of control isotypic IgG and analyzed after 28 days of infection. Bacterial load (**A**), interstitial macrophages (**B**), alveolar macrophages (**C**), B cells (**D**) and cytotoxic T cells (**E**) were measured

by flow cytometric analysis in cell suspensions of murine lungs. Dots represent cells in individual mice, horizontal lines represent mean values and the error bars represent the SEM. The data are pooled from at least two independent experiments (n=8-10). Values represent the mean ± SEM.

Table S1. Characteristics of CF patients enrolled for the study.

**Patients characteristics**	**CF patients (n=55)**
Age (years)*	20,5 (5-36,1)
Gender, male (%)[§]	23 (41.8%)
FEV_1 (% predicted)^	64,3 (21,2-120)
F508del/F508del homozygotes (%)	18 (32,7%)
P. aeruginosa only[§]	15 (27%)
P. aeruginosa and other bacteria[§]	33 (60%)
Colonized < 2 years by *P. aeruginosa*[§]	11 (20%)
Chronically colonized by *P. aeruginosa* > 6 years (%)[§]	26 (47,3%)
No *P. aeruginosa*	18 (32,7%)

Data are expressed as * mean, ^ median or [§] positive events.

Table S2. Levels of IL-17A, IL-4 and IFN-γ in the sera of CF patients intermittently or chronically colonized by *P. aeruginosa*.

	Levels (mean±SEM)	Number of Patients*
**IL-4**	11,90 ± 0,53	27
**IL-17**	5,08 ± 2,11	5
**IFN-γ**	180,80 ± 41,47	25

* patients in whose sera the marker was detectable (28 patients analyzed)

Table S3. Frequencies of T cells subtypes in the spleen of C57Bl/6 mice after 28 days of chronic infection by *P. aeruginosa* AA43 isolate.

	2 days			28 days		
	**Ctrl** (mean ± SEM; n=6)	**AA43** (mean ± SEM; n=4)	**Ctrl vs AA43** (P value)	**Ctrl** (mean ± SEM; n=4)	**AA43** (mean ± SEM; n=12)	**Ctrl vs AA43** (P value)
**Total cells**	$8,42x10^{7}$ ±$1,46x10^{7}$	$3,93x10^{7}$ ± $7,92x10^{6}$	≥ 0,05	$1,2x10^{8}$ ± $1,44x10^{7}$	$1,11x10^{8}$ ± $6,33x10^{6}$	≥ 0,05
			**Frequency/$CD45^{+}$**			
**$CD3^{+}$**	26,03 ± 0,78	27,93 ± 0,69	≥ 0,05	35,33 ± 1,38	31,4 ± 1,9	≥ 0,05
**$CD4^{+}$**	16,37 ± 0,46	16,7 ±0,54	≥ 0,05	21,64 ± 0,65	19,51 ± 1,26	≥ 0,05
**$CD8^{+}$**	8,08 ± 0,38	9,25 ± 0,16	≥ 0,05	11,81 ± 0,71	10,06 ± 0,62	≥ 0,05
			**Frequency/$CD4^{+}$**			
**IL-$17A^{+}$**	0,47 ± 0,19	0,28 ± 0,08	≥ 0,05	0,51 ± 0,14	0,54 ± 0,07	≥ 0,05
**IFN-γ^{+}**	2,13 ± 0,37	1,25 ± 0,15	≥ 0,05	2,34 ± 0,20	2,46 ± 0,21	≥ 0,05
**IL-4^{+}**	0,54 ± 0,14	0,42 ± 0,10	≥ 0,05	0,14 ± 0,08	0,14 ± 0,03	≥ 0,05
			**Frequency/$CD8^{+}$**			
**IL-$17A^{+}$**	0,22 ± 0,11	0,13 ± 0,03	≥ 0,05	0,09 ± 0,03	0,12 ± 0,02	≥ 0,05
**IFN-γ^{+}**	12,54 ± 2,17	9,67 ± 1,7	≥ 0,05	10,21 ± 1,02	15,86 ± 2,08	≥ 0,05
